# Patent foramen ovale closure: A prospective UK registry linked to hospital episode statistics

**DOI:** 10.1371/journal.pone.0271117

**Published:** 2022-07-14

**Authors:** Iain Willits, Kim Keltie, Robert Henderson, Mark de Belder, Nicholas Linker, Hannah Patrick, Helen Powell, Lee Berry, Samuel Urwin, Helen Cole, Andrew J. Sims

**Affiliations:** 1 The Newcastle upon Tyne Hospitals NHS Foundation Trust, Northern Medical Physics and Clinical Engineering, Newcastle, United Kingdom; 2 Translational and Clinical Research Institute, Newcastle University, Newcastle, United Kingdom; 3 Trent Cardiac Centre, Nottingham University Hospitals NHS Trust, Nottingham, United Kingdom; 4 National Institute for Cardiovascular Outcomes Research (NICOR), Barts Health NHS Trust, London, United Kingdom; 5 South Tees Hospitals NHS Foundation Trust, Cardiology, Middlesbrough, United Kingdom; 6 National Institute for Health and Care Excellence, London, United Kingdom; 7 National Institute for Health and Care Excellence, Manchester, United Kingdom; Ohio State University, UNITED STATES

## Abstract

**Aims:**

PFO closure is a percutaneous intervention, which aims to reduce risk of recurrent stroke by preventing paradoxical embolism. The objective of this study was to measure procedural safety and longer-term effectiveness of PFO closure in a UK setting.

**Methods and results:**

Prospective registry data from patients with cryptogenic stroke eligible for PFO closure were collected for up to 2 years and linked to routine data sources for additional follow-up. Outcomes of interest included procedural success rate, health related quality of life, and longer-term death and neurological event rates.

A total of 973 PFO closure procedures in 971 patients were included in analysis. Successful device implantation was achieved in 99.4 [95% CI 98.6 to 99.8]% of procedures, with one in-hospital death. During median follow-up of 758 (Q1:Q3 527:968) days, 33 patients experienced a subsequent neurological event, 76% of which were ischaemic in origin. Neurological event rate was 2.7 [95%CI 1.6 to 3.9]% at 1-year (n = 751) and 4.1 [95% CI 2.6 to 5.5]% at 2-years (n = 463) using Kaplan-Meier analysis. Improvements in patient quality of life (utility and visual analogue scale) were observed at 6-weeks and 6-months follow-up.

**Conclusion:**

Our observational study demonstrates that PFO closure for prevention of recurrent stroke is a relatively safe procedure but in routine clinical practice is associated with a slightly higher risk of recurrent neurological events than in randomised trials. We hypothesize that our study enrolled unselected patients with higher baseline risk, who were excluded from randomised trials, but who may benefit from a similar relative reduction in risk from the intervention.

## Introduction

Each year in the UK, there are more than 100,000 strokes causing 38,000 deaths, [1] which are responsible for about 7% of total mortality in men and 9% in women [2]. Stroke places a substantial financial burden on healthcare services, estimated to account for 5% of National Health Service (NHS) costs in the UK [3]. Approximately 80% of strokes are ischaemic in origin, caused by thrombotic or embolic occlusion of cerebral arteries. In about 25% of ischaemic strokes, the mechanism is uncertain or it is unclear where the embolus originated; these strokes are termed cryptogenic strokes [4].

A patent foramen ovale (PFO) is a common heart finding affecting about 25% of the adult population [5]. For the majority of people, PFO is a clinically inconsequential communication between the right and left atria but in a minority of people the PFO provides a mechanism for paradoxical embolism. The role of PFO in the aetiology of cryptogenic stroke is not fully understood, but a causal relationship has been proposed [6]. Secondary preventative medical management of cryptogenic stroke in patients with PFO usually consists of antiplatelet drugs rather than systemic anticoagulation [7], unless the patient has been identified as having an underlying thrombophilic condition or is at persistent risk of venous embolism. An alternative management strategy is transcatheter PFO closure, a percutaneous procedure with a low risk of complications [8, 9].

The superiority of PFO closure versus medical treatment only for secondary prevention of stroke has been confirmed unequivocally at an aggregate level by a systematic review and meta-analyses of randomised trials [10, 11]. However, clinical effectiveness data on the routine use of PFO closure are scant. In 2013, the NHS England Commissioning through Evaluation (CtE) Programme allowed patients to access PFO closure, whilst prospective safety and efficacy data were collected in a registry designed to contribute to future commissioning decisions. Here we report the safety and effectiveness of PFO closure in patients with a previous cryptogenic stroke enrolled in this registry.

## Methods

### Design and ethics

This was a prospective observational study using a registry to capture characteristics and outcomes of consecutive patients undergoing PFO closure for secondary prevention of cryptogenic stroke. Data were reported using STROBE criteria [12]. Follow-up was scheduled at 6 weeks, 6 months, 1 year, and 2 years for a range of clinical and patient reported outcomes. Patients were also linked to two administrative datasets to validate the registry data and capture longer-term (2-year) mortality and neurological events (including stroke, transient ischaemic attack and reversible ischaemic neurologic deficit) [13].

Patients gave written informed consent to PFO closure as part of usual clinical care. Approvals for data collection, data linkage and analyses were granted by the NHS Health Research Authority Confidentiality Advisory Group Section 251 (Ref: 17/CAG/0153, CAG 10-07(b)/2014) and NHS Digital (Ref: DARS-NIC-151212-B5Z3R).

### Patient and public involvement

The Commissioning through Evaluation steering group included a lay representative.

### Patient selection, follow-up and outcomes

Twenty hospitals across England contributed data. Patient eligibility for the PFO closure procedure was assessed by a multidisciplinary team (MDT) that included cardiologists and stroke physicians at each centre. Patients were required to have had single or multiple ischaemic stroke or transient ischaemic attack (TIA) events with confirmatory brain imaging, and echocardiographic evidence of a PFO with significant right-to-left shunting, either spontaneously or during provocative manoeuvres. Eligibility was confirmed if the MDT considered that the stroke or TIA was likely to be due to paradoxical embolism through the PFO and could not identify any other cause of the ischaemic event.

Eligible patients who provided informed consent underwent PFO closure under local anaesthesia (with or without sedation) or general anaesthesia, on or after 1^st^ October 2014 when Commissioning through Evaluation began. The PFO closure procedure was undertaken with one of three device types: the Abbott Amplatzer range (PFO Occluder, Septal Occluder, or Cribriform); Gore Cardioform Septal Occluder; and the Occlutech Figulla Flex. In each case, the device size was selected to suit anatomical dimensions. Procedural and in-hospital data were collected to determine safety and efficacy. After discharge from hospital, follow-up data were collected during routine outpatient appointments or by telephone. Follow-up was not always undertaken in the treatment centre. Pre-defined outcome measures captured in the registry included device implantation success rates; in-hospital major and minor complications (S1 and S2 Tables). Patient reported outcomes captured during follow-up (S3 Table) included the visual analogue scale (VAS), and health-related quality of life (HRQoL) score via the EuroQol 5-level (EQ-5D-5L) system, converted into utility scores.

### Data linkage

Data from enrolled patients were linked with Hospital Episode Statistics (HES) and Office of National Statistics (ONS) mortality administrative datasets by NHS Digital [14]. Data from HES included all inpatient finished consultant episodes with hospital discharge dates between 1st April 2008 and 1st March 2018. Data from ONS included all deaths reported until 1st March 2018. Records with demographic and administrative details that conflicted between the linked data sources were flagged to indicate potential errors in matching (i.e. matching to an incorrect patient) and excluded from subsequent long-term analysis. Outcomes of interest from data linkage were mortality and neurological (ischaemic and haemorrhagic) events (S3 Table).

### Statistics

Data analysis and statistical tests were carried out using the programming language R [15]. Paired quality of life scores, utilities and medication recorded in the registry were compared at each follow-up point against baseline using Fisher’s exact tests or t-tests where appropriate. Kaplan-Meier analysis was applied to mortality outcomes from linked data and event rates reported at 1 and 2-year time points.

## Results

In all, 1174 unique procedure records from 1170 patients were recorded in the registry between October 2014 and August 2017, Fig 1. A total of 201 procedure records were excluded from further analysis, including 145 procedures which did not include previous stroke(s) or TIA(s) as a reason for treatment. This left 973 eligible procedures from 971 patients (2 patients had a second PFO closure procedure at 11 and 7 months after first procedure, for a residual interatrial communication detected during follow-up).

**Fig 1:**
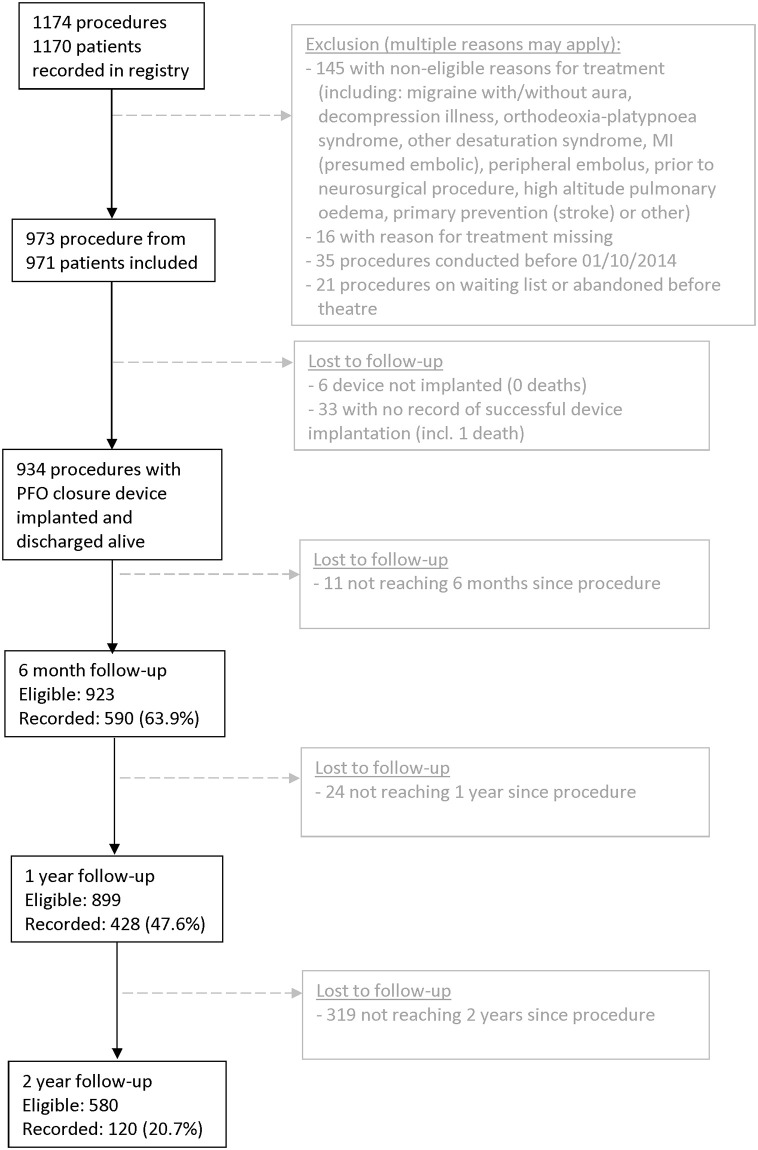
Patient flow in the CtE registry. Outcomes (stroke, death, TIA) were derived from data linkage.

Baseline characteristics for each procedure are reported in Table 1. The median age was 45 years (range 17 to 82 years). Aortic arch imaging was available for 214 patients and was normal in 212 patients (grade 1) and showed diffuse intimal thickening (grade 1) in 2 patients. The median PFO tunnel length was 6 mm (range 1 to 20 mm) and the diameter was 9 mm (range 1 to 30 mm). There was widespread use of anti-thrombotic medication, with most receiving antiplatelet drugs (82%) and 12% receiving an anticoagulant prior to the procedure.

**Table 1:** Procedural characteristics and investigations.

	PFO closure procedures (n = 973)*
Female, n (%)	419 (43.1%)
Age, years	45
median (Q1,Q3) [range]	(36,51) [17–82]
Risk factors, n (%):	
Diabetes	26 (2.8%)
Hypertension	102 (11.1%)
Hyperlipidaemia	159 (17.6%)
Prior myocardial infarction	20 (2.2%)
Peripheral vascular disease	4 (0.4%)
Previous venous thrombosis/thromboembolic disease	41 (4.6%)
Thrombophilic condition	30 (4.7%)
History of arrhythmia	24 (2.6%)
CHA_2_DS_2_-VASc score	
2	379 (39.0%)
3	373 (38.3%)
4	39 (4.0%)
5	14 (1.4%)
6	1 (0.6%)
Not recorded	167 (17.2%)
Atrial septal aneurysm, n (%)	88 (9.9%)
Brain scan (MRI/CT)^&^, n (%):	
Not conducted†	41 (5.2%)
Conducted, no ischaemic lesion	74 (9.3%)
Conducted, ischaemic lesion	678 (85.5%)
Pre-procedural PFO assessment method, n (%):	
TTE (colour–flow mapping or bubble contrast)	472 (51.3%)
TOE (colour–flow mapping or bubble contrast)	166 (18.0%)
Transcranial Doppler	3 (0.3%)
Combination	279 (30.4%)
R-to-L shunt detected, n (%)	817 (96.8%)
Echo contrast R-to-L shunt (without provocation), n (%):	
None	115 (16.5%)
Individual bubble (<5 per still frame)	114 (16.4%)
Clusters/clouds/chamber opacification (≥ 5 per still frame)	467 (67.1%)
Echo contrast R-to-L shunt (with provocation), n (%):	
None	12 (1.8%)
Individual bubble (<5 per still frame)	8 (1.2%)
Clusters/clouds/chamber opacification (≥ 5 per still frame)	647 (97.0%)
Cerebro-vascular imaging (by carotid ultrasound scan or MR/CT angiography), n (%):	
Not done	150 (19.3%)
Normal	597 (76.6%)
Minor abnormality	22 (2.8%)
Moderate/severe lesion	10 (1.3%)
Aortic atheroma in arch, n (%):	
Not imaged	619 (74.3%)
Grade 1 (Normal appearance)	212 (25.5%)
Grade 2 (Diffuse intimal thickening)	2 (0.2%)
Grade 3 (Sessile plaque protruding < 5mm into aorta)	0 (0.0%)
Grade 4 (Sessile plaque protrucing ≥ 5mm into aorta)	0 (0.0%)
Medication (pre-procedure), n (%):	
Single antiplatelet	560 (60.5%)
Dual antiplatelet	167 (18.1%)
Anticoagulant alone	83 (9.0%)
Combined antiplatelet/anticoagulant	27 (2.9%)
Other	66 (7.1%)
None	22 (2.4%)
PFO tunnel length (mm), median (Q1,Q3) [range]	6 (3,10) [1–20]
Max PFO diameter (mm), median (Q1,Q3) [range]ⱡ	9 (6,12) [1–30]

Abbreviations: CHA_2_DS_2_-VASc score, clinical prediction rule for estimating risk of stroke; CT, computed tomography; MRI, magnetic resonance; TOE, transoesophageal echocardiogram; TTE, transthoracic echocardiogram.

*Not all data fields were complete for every patient at baseline and follow-up. The percentages presented in this table were calculated using the number of patients with each characteristic reported as the denominator.

^&^All patients were reviewed by an MDT.

^†^Not thought necessary or applicable, not available in appropriate timeframe, not available at this hospital

^ⱡ^ Assessed by echo or fluoroscopy

Procedural information is reported in Table 2. Attempted deployment of 953 named devices was recorded in the registry (with 2 devices attempted in 26 patients, 3 devices attempted in 4 patients); PFO closure was attempted with an Amplatzer device in 55% of procedures. The data field for device implantation success was completed for 940 procedures, with successful device implantation in 934 procedures (99.4 [95% CI 98.6 to 99.8]%); 6 confirmed failures to deploy the device (3 unable to position correctly, 1 incorrect size, 1 complication, 1 other reason with no further detail provided). The procedural success rate (successful implant without major complication) was 95.4 [93.9 to 96.6]%. Eight procedures (0.8 [0.4 to 1.6]%) had an in-hospital major complication, including one death (due to multi-organ failure associated with fungal endocarditis) and three neurological events, S3 Table. There were 23 minor in-hospital complications (including 9 patients who developed atrial fibrillation; 5 required treatment, and 4 reverted spontaneously to sinus rhythm), making a total procedural complication rate of 2.4% [1.5 to 3.5]%, S4 Table.

**Table 2:** Procedural details and in-hospital complications of people included in the registry.

	PFO closure procedures (n = 973)*
Anaesthesia, n (%):	
General	700 (74.9%)
Local with sedation	160 (17.1%)
Local only	75 (8.0%)
Intra-operative echo imaging, n (%):	
TOE (planned) or TTE)	694 (73.6%)
ICE planned	208 (22.1%)
ⱡUnplanned (TOE/ICE)	10 (1.1%)
None	31 (3.3%)
Device, n(%):	
Abbot (Amplatzer range)	523 (54.9%)
GORE (Cardioform Septal Occluder)	288 (30.2%)
Occlutech (Figulla Flex)	121 (12.7%)
Other (incl. combination)	21 (2.2%)
Procedural duration (mins), median (Q1:Q3) [range]	45 (30,60) [0–229]
**Major complications, n (%)**	8 (0.8% [95% CI 0.4 to 1.6]%)
**Minor complication, n (%)**	23 (2.4% [95% CI 1.5 to 3.5]%)
**Any complication, n (%)**	**30 (3.3%, [95% CI 2.2:4.6]); from 970 available** *
**Device implanted, n (%)**	**934 (99.4%, [95% CI 98.6:99.8]), from 940 available** *
**Procedural success** † **, n (%)**	**928 (95.4%, [95% CI 93.9:96.6]), from 973 available** *

Abbreviations: CI, 95% confidence interval; ICE, intracardiac echocardiography; TOE, transoesophageal echocardiogram; TTE, transthoracic echocardiogram.

* Not all data fields were complete for every patient at baseline and follow-up. The percentages presented in this table were calculated using the number of patients with each characteristic reported as the denominator.

^†^ Defined as device implanted successfully in absence of major complications.

^ⱡ^ Unplanned at start of procedure

### Follow-up

**Fig 2:**
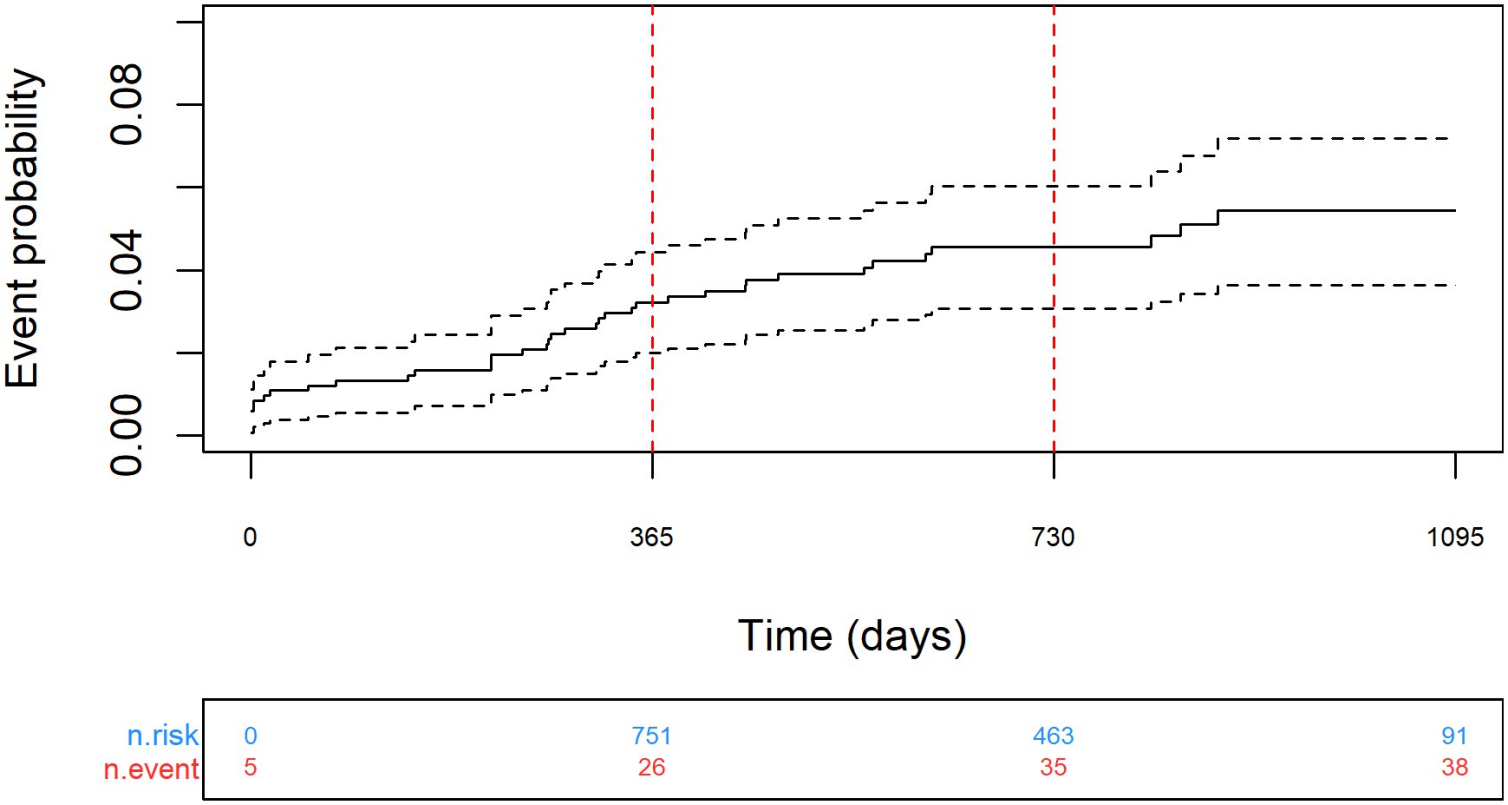
Kaplan-Meier analysis of mortality or neurological event over 2 years’ follow-up (dotted lines show 95% confidence intervals).

A total of 840 patients from the registry were matched to HES/ONS (92% successful matching rate after additional cleaning, S1 Fig) for analysis of long-term safety and efficacy, resulting in median [IQR] follow-up of 2.2 [1.7 to 2.7] years. Procedural follow-up was recorded in the registry in 75.5% of cases at 6 weeks, 63.9% at 6 months, 47.6% at 1 year and 20.7% at 2 years. No embolization or malposition during follow-up was reported in the registry. Four patients required additional cardiac intervention, including 2 who underwent percutaneous intervention with another device, 1 patient who underwent surgical closure and 1 undefined. From analysis of registry and linked data (S4 Table), 33 patients (3.9%, 33/840) had a neurological event following the PFO closure procedure. Most of these (25/33, 76%) were of ischaemic origin, 7 (21%) were of unknown type, and one person (3%) had a haemorrhagic stroke, Table 3. The Kaplan-Meier combined event rates (neurological event or death) at 1 and 2-years were 3.2 [95% CI 2.0 to 4.4]% (n = 751) and 4.6 [95% CI 3.1 to 6.0]% (n = 463) respectively, Fig 2. Neurological event and mortality rates are reported separately within S2 and S3 Figs respectively.

**Table 3:** Longitudinal outcomes: All-cause mortality, neurological events and composite outcome from linked data (registry and HES).

	All-cause mortality	Total neurological events	Total neurological events combined with all-cause mortality	Ischaemic neurological events
No. of events	7	33	38	25
Mean (SD) follow-up, days	784 (257)	736 (302)	734 (303)	734 (303)
Median [Q1:Q3] follow-up, days	803 [603:989]	760 [528:975]	758 [527:968]	758 [527:968]
Unadjusted event rate, per 100 person years follow-up (95% CI)	0.4 (0.2 to 0.8)	2.0 (1.3 to 2.7)	2.3 (1.6 to 3.1)	1.5 (1.0 to 2.2)
1-year event-free probability (95% CI) [number at risk]	99.5 [99.0 to 100.0]% (n = 800)	97.3 [96.1 to 98.4]% (n = 751)	96.8 [95.6 to 98.0]% (n = 751)	97.8 [96.7 to 98.8]% (n = 751)
2-year event-free probability (95% CI) [number at risk]	99.2 [98.6 to 99.9]% (n = 504)	95.9 [94.5 to 97.4]% (n = 463)	95.4 [94.0 to 96.9]% (n = 463)	96.8 [95.6 to 98.1]% (n = 463)

Abbreviations: CI, confidence interval.

The mean (standard deviation, SD) baseline HRQoL utility score (n = 432) was 0.87 (0.19). Using paired analysis, a statistically significant increase in HRQoL significantly was observed at 6 weeks (n = 242 pairs; mean change of 0.03, SD = 0.16, p = 0.0185), which was sustained until 6 months (n = 210 pairs, mean change of 0.03, SD = 0.17, p = 0.0047); utility scores numerically improved in 32%, did not change in 45% and numerically decreased in 23% of pairs, S5a Table. Patient assessment of baseline health (measured using visual analogue scale, VAS) had a median score of 80 (Q1:Q3, 70:90)(n = 365 patients). A statistically significant increase in VAS was also observed at 6 weeks (mean increase 4.8 (SD 14.0) in 199 pairs, p<0.0001) and 6 months (mean increase 6.0 (SD 16.8) in 167 pairs, p<0.0001), S5b Table. These changes were mainly due to improvements in the anxiety and depression domain, with significant improvements relative to baseline at 6 weeks (n = 242, p = 0.008) and at 6 months (n = 210, p = 0.01) with no significant changes observed in the pain, usual activities, self-care, or mobility domains, S5c Table. There was a significant change in use of medication between baseline and discharge (n = 863, p<0.001) with increased use of antiplatelets and decrease in anticoagulants; but no significant changes during follow-up, Table 4.

**Table 4:** Medication use over time in patients with device implanted and completed follow-up compared with baseline (using available paired data). p-values from 3x2 Fisher’s exact tests.

	Medication at baseline (pre-procedure)						
	Antiplatelet only	Anti-coagulant	Other (incl. None)	Antiplatelet only	Anti-coagulant	Other (incl. None)	p-value
Discharge (n = 863)	697 (80.8%)	88 (10.2%)	78 (9.0%)	766 (88.8%)	48 (5.6%)	49 (5.7%)	p<0.0001
6 weeks (n = 632)	527 (83.4%)	27 (4.3%)	78 (12.3%)	527 (83.4%)	33 (5.2%)	72 (11.4%)	0.65
6 months (n = 531)	436 (82.1%)	17 (3.2%)	78 (14.7%)	436 (82.1%)	27 (5.1%)	68 (12.8%)	0.23
1 year (n = 350)	262 (74.9%)	10 (2.9%)	78 (22.3%)	262 (74.9%)	16 (4.6%)	72 (20.6%)	0.46
2 years (n = 104)	70 (67.3%)	0 (0%)	34 (32.7%)	70 (67.3%)	4 (3.8%)	30 (28.8%)	0.15

## Discussion

This study reports the safety and efficacy of PFO closure for secondary prevention of stroke from a multicentre, prospective, observational registry, with patient selection and treatment reflecting routine practice within the NHS in England. The results of this registry study, along with a review of published evidence informed NHS England’s decision to commission PFO closure routinely [16].

The key findings of our study include a technical success rate of PFO closure in excess of 99.4% and a major in-hospital complication rate of 0.8% with 1 death (endocarditis) reported, and 3 cases of neurological event (1 cerebrovascular accident/ reversible ischaemic neurological deficit, 1 ischaemic and 1 of undetermined origin). The neurological event rate, the key efficacy outcome measured using Kaplan-Meier analysis, was 2.7% after 1 year and 4.1% after 2 years. Most neurological events were ischaemic in origin. Statistical improvements in VAS and HRQoL were observed at 6 weeks and 6 months follow-up.

This study was single-armed and did not report comparative data. It was not the prospective intention of this study to investigate differences in outcome by technology or treating hospital, and outcomes may have been influenced by learning curve. The CHA_2_DS_2_-VASc score of our cohort (median of 3; 806 with a recorded score, Table 1) predicts an ischaemic stroke rate at 1-year of 3.7% [17], which is higher than the observed ischemic event rate of 2.2 [1.2 to 3.3]% but this comparison is confounded as the CHA_2_DS_2_-VASc score predicts stroke rates for people with a different risk factor (atrial fibrillation); only 2.6% of our cohort had previous history of arrhythmia. We acknowledge that the ROPE score, developed from pooled data on 3674 patients, can be used to predict the likelihood that a patient with cryptogenic stroke has a PFO [18]. Validation studies of the score are limited, however, it is not currently used in routine clinical practice in the United Kingdom, and it was not included in the design of our study. A recent retrospective analysis of data from three randomized trials reported an association between the ROPE score and the impact of PFO closure on the risk of recurrent stroke, but the authors concluded that analysis of larger datasets will be required to determine the role of the ROPE score in clinical decision-making [19].

Three randomised trials reported event rates at 2-years: the event rate in the closure group of the RESPECT trial [20] was 1.6%; the CLOSE (n = 238) [21] and DEFENCE-PFO (n = 60) [22] trials both reported 0% event rates; all lower than our study (4.1 [2.6 to 5.5]%). The CLOSURE-1 trial reported an event rate of 5.8% in the per-protocol population for a composite outcome of stroke or TIA in the intervention arm, however the STARFlex device used in the CLOSURE-1 trial is no longer available for clinical use [23]. Other relevant randomised trials (PC [24], REDUCE [25]) did not report Kaplan-Meier rates at 1 or 2 years in their intervention arms and cannot be directly compared with our study, S6 Table. Differences in event rates could be a consequence of loss to follow-up in the registry, however this was ameliorated in our study by linkage to national routine datasets (HES, ONS). Event rates may also differ as a direct consequence of study design; RCTs generally have strict recruitment criteria, which may favour selection of patients with lower baseline risk of the endpoint. By contrast, registries generally have broader inclusion criteria and are more likely to be representative of routine clinical practice, enrolling patients across a broad spectrum of baseline risk. Furthermore, TIA and reversible ischaemic neurological deficit (RIND) were included in our definition of neurological events but not included in the endpoint definitions of the randomised trials, S6 Table. Of note, in a subgroup of the DEFENSE trial 8.8% of patients in the PFO group were found to have ‘silent’ brain infarction on follow-up magnetic resonance brain imaging [10].

One strength of the CtE registry was that it reported HRQoL outcomes. These data suggest that the procedure was associated with a reduction in anxiety and depression, at least in the short-term; we speculate that patients with stroke/TIA attributed to paradoxical embolism may have increased anxiety about the risk of recurrent stroke that improves when the PFO is successfully closed. This manifested as an overall improvement in HRQoL, with the measured change (0.03) near the lower end of range of estimates of the minimum clinically important difference in HRQoL [26]. However, it is likely that patient numbers and follow-up are insufficient to detect changes in quality of life associated with recurrence of neurological events that might influence the cost-effectiveness of these technologies, which has not yet been established in a UK setting. Currently there appears to be a deficit in patient-orientated outcomes in this field, which may warrant further research.

## Conclusions

In conclusion, this was a large prospective, observational study on the safety and efficacy of PFO closure in the UK. Our study suggests that PFO closure can be done safely in routine practice and the relatively low rates of neurological events during follow-up suggest that the therapeutic benefit of PFO closure seen in the RCTs is also likely to be seen in routine UK practice. We hypothesize that our study enrolled unselected patients with higher baseline risk, who were excluded from randomised trials, but who may benefit from a similar relative reduction in risk from the intervention. Further research is required to identify the patients for whom PFO closure is most likely to be cost-effective in the NHS.

## Supporting information

S1 Table Definition of in-hospital major complications. (DOCX)

S2 Table Definition of in-hospital minor complications. (DOCX)

S3 Table Definition of long-term outcomes. (DOCX)

S4 Table Additional details of major and minor in-hospital complications recorded in the registry. (DOCX)

S5 Table Change in a) utility, b) visual analogue scale (VAS), c) EQ5D domains at follow-up when compared with pre-procedure at follow-up. (DOCX)

S6 Table Comparison of demographics, inclusion and exclusion criteria between the RCTs (intervention arms) and this study. (DOCX)

S1 Fig Data flow describing linkage to HES. (DOCX)

S2 Fig Kaplan-Meier analysis of mortality over 2 years follow up. (TIFF)

S3 Fig Kaplan-Meier analysis of neurological events over 2 years follow up. (TIFF)
