# Supplementary material for: Patent foramen ovale closure: A prospective UK registry linked to hospital episode statistics
Source: PLoS One. 2022 Jul 14;17(7):e0271117. doi: 10.1371/journal.pone.0271117 (PMC9282467; doi:10.1371/journal.pone.0271117)
Supplement: S1 Table — (DOCX) [file pone.0271117.s001.docx]

Table S1: Definition of in-hospital major complications

| Outcome | Description |
| --- | --- |
| Death | In-hospital death following/during procedure |
| Neurological event | Stroke (ischaemic, haemorrhagic, undetermined)  CVA/RIND  TIA |
| Embolisation | Device embolization including percutaneous retrieval, surgical retrieval and not retrieved. |
| Major vascular problems | Vascular surgical repair  Vascular stent or other percutaneous procedure  Any other cardiac surgical procedure |
| Cardiac structural complication | Atrial of any cardiac perforation  Device erosion without haemodynamic consequence  Any new cardiac fistula (e.g. aorta to right atrium)  Cardiac tamponade requiring surgical or percutaneous drainage  Pericardial effusion requiring surgical or percutaneous drainage |
| Major bleed | Fatal bleeding (BARC type 5) OR  Bleeding in a critical organ, such as intracranial, intraspinal, intraocular, or pericardial necessitating pericardiocentesis, or intramuscular with compartment syndrome (BARC type 3b and 3c) OR  Bleeding causing hypovolemic shock or severe hypotension requiring vasopressors or surgery (BARC type 3b) OR  Overt source of bleeding with drop in haemoglobin of ≥5 g/dL or whole blood or packed red blood cells (RBCs) transfusion ≥4 units* (BARC type 3b) OR  Major bleeding (BARC type 3a); Over bleeding either associated with a drop in the haemoglobin level of at least 3.0 g/dL or requiring transfusion of 2 or 3 units of whole blood/RBC AND does not meet criteria of life-threatening or disabling bleeding. |
| Additional surgery | Device embolization with surgical retrieval  Cardiac tamponade requiring surgical drainage  Pericardial effusion requiring surgical drainage |
| Other | Myocardial infarction  Endocarditis  Oesophageal rupture |
| Abbreviations: BARC Bleeding Academic Research Consortium; CVA cerebrovascular accident; RIND reversible ischaemic neurologic deficit; TIA transient ischaemic attack; | |
