# Supplementary material for: Patent foramen ovale closure: A prospective UK registry linked to hospital episode statistics
Source: PLoS One. 2022 Jul 14;17(7):e0271117. doi: 10.1371/journal.pone.0271117 (PMC9282467; doi:10.1371/journal.pone.0271117)
Supplement: S2 Table — (DOCX) [file pone.0271117.s002.docx]

Table S2: Definition of in-hospital minor complications

| Outcome | Description |
| --- | --- |
| Device malfunction | Device malposition after release (corrected, or not corrected)  Device malfunction probably iatrogenic |
| New or worsening AF | New or worsening AF or other atrial tachyarrhythmia (spontaneous correction, or treatment required) |
| Other arrhythmia | VT/VF requiring treatment  Temporary pacing  Permanent pacing |
| Cardiac structural complication | Pericardial effusion (no drainage)  Other cardiac structural complications (during procedure or prior to discharge) |
| Peripheral embolic event | Peripheral embolic event  Thrombus detected in LA or on device |
| Vascular problems | Bleeding problem  Blood transfusion  AV fistula  Pseudoaneurysm  Major haematoma |
| Minor bleed | Minor bleeding (BARC type 2 or 3a, depending on the severity)  Any bleeding worthy of clinical mention (e.g. access site haematoma) that does not qualify as life-threatening, disabling, or major |
| Other | Air embolism  Migraine or worsening migraine  Oesophageal trauma – haematemesis  Nickel allergy  Transient ST elevation no MI |
| Abbreviations: AF atrial fibrillation; BARC Bleeding Academic Research Consortium; MI myocardial infarction; LA left atrium; VF ventricular fibrillation; VT ventricular tachycardia | |
