# Supplementary material for: Patent foramen ovale closure: A prospective UK registry linked to hospital episode statistics
Source: PLoS One. 2022 Jul 14;17(7):e0271117. doi: 10.1371/journal.pone.0271117 (PMC9282467; doi:10.1371/journal.pone.0271117)
Supplement: S3 Table — (DOCX) [file pone.0271117.s003.docx]

Table S3: Definition of long-term outcomes

| Outcome | Description |
| --- | --- |
| Neurological event by 1 year | A registry field completed prior to discharge or at follow-up with options: none, yes-ischaemic, yes-haemorrhagic, yes-undetermined (which did not differentiate CVA from TIA); or  A registry field completed prior to discharge or at follow-up with options: CVA/RIND, TIA, other, unknown; or  In HES, an admission recorded with any of the diagnostic codes:  I60 Subarachnoid haemorrhage  I61 Intracerebral haemorrhage  I62 Other non-traumatic intracranial haemorrhage  I63 Cerebral infarction  I64 Stroke, not specified as haemorrhage or infarction  G45.8 Other transient cerebral ischaemic attacks and related syndromes  G45.9 Transient cerebral ischaemic attack, unspecified  Note that neither the registry nor HES stipulate that the CVA or TIA event had to be verified by imaging. |
| Mortality | In the registry, recorded as status at discharge (alive/dead) and at follow-up including date and cause of death (if known).  Record within ONS mortality dataset with death and cause of death (if reported). |
| Visual Analogue Scale | A registry field for patient-reported health-related quality of life captured by the visual analogue rating scale. |
| EuroQol 5D 5L | Registry fields capturing 5 dimensions (mobility, self-care, usual activities, pain/discomfort, anxiety/depression) at baseline and follow-up. |
| Health-related quality of life | Conversion of EuroQol 5D 5L into utility scores. |
| Medication | A registry field, recorded at discharge and at follow-up, with the following options (multiple choices permitted):  No antiplatelet or oral anticoagulant treatment  Aspirin  Clopidogrel  Prasugrel  Ticagrelot  Dipyrimadole  Cilastazol  Warfarin  Other oral vitamin K antagonist  Dabigatran  Apixaban  Rivaroxaban  Low molecular weight heparin  Other  Unknown/Not applicable |
| Abbreviations: CVA cerebrovascular accident; HES Hospital Episodes Statistics; ONS, Office of National Statistics; TIA transient ischaemic attack. | |
