# Supplementary material for: Patent foramen ovale closure: A prospective UK registry linked to hospital episode statistics
Source: PLoS One. 2022 Jul 14;17(7):e0271117. doi: 10.1371/journal.pone.0271117 (PMC9282467; doi:10.1371/journal.pone.0271117)
Supplement: S4 Table — (DOCX) [file pone.0271117.s004.docx]

Table S4: Additional details of major and minor in-hospital complications recorded in the registry.

|  | **PFO closure procedures (n=973)*** |
| --- | --- |
| **Major complications** | 8 (0.8% [95% CI 0.4 to 1.6]% |
| Death | 1 (0.1, 0.0:0.6) |
| Neurological event | 3 (0.3, 0.0:0.9) |
| Device embolisation | 3 (0.3, 0.1:0.9) |
| Major cardiac structural complications | 0 (0.0, 0.0:0.4) |
| MI | 1 (0.1, 0.0:0.6) |
| Major vascular injury | 0 (0.0, 0.0:0.4) |
| Endocarditis | 0 (0.0, 0.0:0.4) |
| Oesophageal rupture | 0 (0.0, 0.0:0.4) |
| Major bleed | 1 (0.1, 0.0:0.6) |
| Additional surgery | 1 (0.1, 0.0:0.6) |
| **Minor complication** | 23 (2.4% [95% CI 1.5 to 3.5]% |
| Device malfunction | 0 (0.0, 0.0:0.4) |
| Air embolism | 0 (0.0, 0.0:0.4) |
| Malposition | 4 (0.4, 0.1:1.1) |
| New/worsening AF | 9 (1.0, 0.4:1.8) |
| Other arrhythmias | 0 (0.0, 0.0:0.4) |
| Minor cardiac structural complications | 0 (0.0, 0.0:0.4) |
| Transient ST elevation (no MI) | 0 (0.0, 0.0:0.4) |
| Minor embolic events | 0 (0.0, 0.0:0.4) |
| Minor vascular injury | 5 (0.5, 0.2:1.2) |
| Migraine/worsening migraine | 3 (0.3, 0.1:0.9) |
| Oesophageal trauma | 0 (0.0, 0.0:0.4) |
| Nickel allergy | 0 (0.0, 0.0:0.4) |
| Minor bleed | 5 (0.5, 0.2:1.2) |
| **Any complication (minor & major combined)** | **30 (3.3, 2.2:4.6)** |
| **Device implanted** | **934 (99.4, 98.6:99.8)** |
| **Procedural success†** | **928 (95.4, 93.9:96.6)** |
| Abbreviations: AF, atrial fibrillation; CI, 95% confidence interval; MI, myocardial infarction  * Not all data fields were complete for every patient at baseline and follow-up. The percentages presented in this table were calculated using the number of patients with each characteristic reported as the denominator.  † Defined as device implanted successfully in absence of major complications. | |
