# Supplementary material for: Patent foramen ovale closure: A prospective UK registry linked to hospital episode statistics
Source: PLoS One. 2022 Jul 14;17(7):e0271117. doi: 10.1371/journal.pone.0271117 (PMC9282467; doi:10.1371/journal.pone.0271117)
Supplement: S5 Table — (DOCX) [file pone.0271117.s005.docx]

Table S5: Change in a) utility, b) visual analogue scale (VAS), c) EQ5D domains at follow-up when compared with pre-procedure at follow-up.

| 5a) | **Utility** | | | |
| --- | --- | --- | --- | --- |
|  | 6 weeks | 6 months | 1 year | 2 years |
| Pairs | 242 | 210 | 129 | 36 |
| Mean (SD) change in utility | 0.06 (0.16) | 0.03 (0.17) | 0.01 (0.20) | -0.01 (0.16) |
| p-value | 0.0185 | 0.0047 | 0.4746 | 0.6582 |
| Improvement | 81 (33.5%) | 75 (35.7%) | 41 (31.8%) | 7 (19.4%) |
| Deterioration | 40 (16.5%) | 37 (17.6%) | 30 (23.3%) | 9 (25.0%) |
| No change | 121 (50.0%) | 98 (46.7%) | 58 (45.0%) | 20 (55.6%) |

| 5b) | **VAS** | | | |
| --- | --- | --- | --- | --- |
|  | 6 weeks | 6 months | 1 year | 2 years |
| Pairs | 199 | 167 | 100 | 23 |
| Mean (SD) change  In VAS | 4.8 (14.0) | 6.0 (16.8) | 2.3 (18.0) | 5.4 (14.3) |
| p-value | <0.0001 | <0.0001 | 0.20 | 0.08 |
| Improvement | 121 (60.8%) | 121 (72.5%) | 51 (51%) | 14 (60.9%) |
| Deterioration | 31 (15.6%) | 32 (19.2%) | 34 (34%) | 6 (26.1%) |
| No change | 47 (23.6%) | 33 (19.8%) | 15 (15%) | 3 (13.0%) |

| 5c) | **Anxiety/Depression** | | | |
| --- | --- | --- | --- | --- |
|  | 6 weeks  (n=242) | | 6 months  (n=210) | |
|  | Baseline | 6 weeks | Baseline | 6 weeks |
| 0. I am not anxious or depressed | 148 | 184 | 121 | 155 |
| 1. I am slightly anxious or depressed | 59 | 38 | 54 | 35 |
| 2. I am moderately anxious or depressed | 28 | 15 | 26 | 14 |
| 3. I am severely anxious or depressed | 3 | 2 | 5 | 3 |
| 4. I am extremely anxious or depressed | 4 | 3 | 4 | 3 |
| Fisher’s test p-value | 0.0085 | | 0.0112 | |

|  | **Pain** | | | |
| --- | --- | --- | --- | --- |
|  | 6 weeks  (n=242) | | 6 months  (n=210) | |
|  | Baseline | 6 weeks | Baseline | 6 weeks |
| 0. I have no pain or discomfort | 198 | 205 | 167 | 176 |
| 1. I have slight pain or discomfort | 32 | 25 | 35 | 24 |
| 2. I have moderate pain or discomfort | 9 | 7 | 6 | 8 |
| 3. I have severe pain or discomfort | 3 | 4 | 1 | 1 |
| 4. I have extreme pain or discomfort | 0 | 1 | 1 | 1 |
| Fisher’s test p-value | 0.7215 | | 0.6132 | |

|  | **Usual activities** | | | |
| --- | --- | --- | --- | --- |
|  | 6 weeks  (n=242) | | 6 months  (n=210) | |
|  | Baseline | 6 weeks | Baseline | 6 weeks |
| 0. I have no problems with performing my usual activities | 195 | 204 | 163 | 179 |
| 1. I have slight problems with performing my usual activities | 31 | 20 | 30 | 16 |
| 2. I have moderate problems with performing my usual activities | 12 | 13 | 14 | 8 |
| 3. I have severe problems with performing my usual activities | 3 | 2 | 2 | 4 |
| 4. I am unable to perform my usual activities | 1 | 3 | 1 | 3 |
| Fisher’s test p-value | 0.4351 | | 0.0707 | |

|  | **Self care** | | | |
| --- | --- | --- | --- | --- |
|  | 6 weeks  (n=242) | | 6 months  (n=210) | |
|  | Baseline | 6 weeks | Baseline | 6 weeks |
| 0. I have no problems with washing or dressing myself | 219 | 225 | 190 | 192 |
| 1. I have slight problems with washing or dressing myself | 19 | 12 | 16 | 13 |
| 2. I have moderate problems with washing or dressing myself | 3 | 4 | 4 | 3 |
| 3. I have severe problems with washing or dressing myself | 1 | 1 | 0 | 2 |
| Fisher’s test p-value | 0.6291 | | 0.6004 | |

|  | **Mobility** | | | |
| --- | --- | --- | --- | --- |
|  | 6 weeks  (n=242) | | 6 months  (n=210) | |
|  | Baseline | 6 weeks | Baseline | 6 weeks |
| 0. I have no problems walking about | 205 | 213 | 175 | 182 |
| 1. I have slight problems walking about | 25 | 15 | 22 | 17 |
| 2. I have moderate problems walking about | 10 | 11 | 11 | 7 |
| 3. I have severe problems walking about | 1 | 3 | 2 | 4 |
| 4. I am unable to walk about | 1 | 0 | 0 | 0 |
| Fisher’s test p-value | 0.3238 | | 0.5198 | |
