# Supplementary material for: Patent foramen ovale closure: A prospective UK registry linked to hospital episode statistics
Source: PLoS One. 2022 Jul 14;17(7):e0271117. doi: 10.1371/journal.pone.0271117 (PMC9282467; doi:10.1371/journal.pone.0271117)
Supplement: S6 Table — (DOCX) [file pone.0271117.s006.docx]

Table S6: Comparison of demographics, inclusion and exclusion criteria between the RCTs (intervention arms) and this study.

|  | CLOSURE-1^21^  (n=447^*^) | RESPECT^18^  (n=499^*^) | PC Trial^22^  (n=204^*^) | CLOSE^19^  (n=238^*^) | Gore REDUCE^23^  (n=441^*^) | DEFENCE-PRO^20^  (n=60^*^) | This study  (Cohort, n=973 procedures, 971 patients) |
| --- | --- | --- | --- | --- | --- | --- | --- |
| *Inclusion criteria* | | | | | | | |
| Age | Between 18 and 60 years old | Between 18 and 60 years old | Less than 60 years | Between 16 and 60 years old | 18 to 59 years old |  | No age limit applied (range 17 to 81 years) |
| History | Previous stroke or TIA within previous 6 months | Randomisation had to occur within 270 days after cryptogenic ischaemic stroke | Not reported | Ischaemic stroke within previous 6 months | Cryptogenic ischaemic stroke or TIA when 180 days of randomisation | Cryptogenic ischaemic stroke within previous 6 months | Prior stroke or TIA confirmed by brain imaging (no time limit applied). Considered by MDT to be highly likely due to right-to-left intra-cardiac shunting. |
| PFO | Documented by TOE with bubble showing right-to-left shunt at the atrial level during a Valsalva manoeuvre | TOE evidence of infused microbubbles in the left atrium within three cardiac cycles after their appearance in the right  atrium, at rest or during Valsalva release. | Documented by TOE | Documented by TTE/TOE | Documented by TTE with bubble at rest or during Valsalva manoeuvre | Documented by TTE with bubble at rest or during Valsalva manoeuvre | Documented echocardiographically by TTE/TOE, with bubble contrast and/or provocative manoeuvres. |
| *Exclusion criteria* | | | | | | | |
| History | Any identified potential cause of ischaemic stroke or TIA other than PFO including carotid-artery stenosis, complex aortic-arch atheroma, clinically significant left ventricular dysfunction or left ventricular aneurysm, AF | Mechanism for the index stroke other than paradoxical embolization could be identified, such as large-vessel disease, any cardioembolic source, a lacunar infarct that was probably due to intrinsic small-vessel disease, or an arterial hypercoagulable state (as indicated by the presence of anticardiolipin antibody, lupus anticoagulant, or hyperhomocysteinemia). | Other identifiable cause of stroke or peripheral thromboembolism (mural thrombus, dilated cardiomyopathy, prosthetic heart valve, mitral stenosis, endocarditis, cardiac myxoma, atherosclerosis or dissection of the aorta, AF, clinically relevant atherosclerosis or dissection of the intra- and extracranial arteries, pre-existing neurological disorder, significant intracranial disease, significant collagen vascular disease, giant cell arteritis, vasculitis, systemic necrotizing vasculitis, hyperviscosity syndroms, hypercoagulable states, contraindication for chronic oral anticoagulant oral antiplatelet therapy, previous surgical or percutaneous PFO closure, severe central nervous system disease, follow-up over next 5 years not possible. | Other identifiable cause of stroke. Previous surgical or endovascular treatments of PFO or ASA. Follow-up impossible or expected poor compliance. Indication for long-term anticoagulant or antiplatelet therapy for another reason. Contraindication to aspirin, clopidogrel, or oral anticoagulants. Increased bleeding risk: severe liver failure, active peptic ulcer, proliferative diabetic retinopathy, history of severe bleeding, history of bleeding or coagulopathy, work or sports activities associated with high-risk of injury and bleeding. | Other identifiable causes of stroke (including large-artery atherosclerotic disease, established cardioembolic source, small-vessel occlusive disease (lacunar stroke), hypercoagulable disorder requiring anticoagulation or arterial dissection). Previous MI, uncontrolled diabetes, uncontrolled hypertension, autoimmune disease, recent history of alcohol or drug abuse, or specific indication for anticoagulation. | Other identifiable causes of stroke (including large artery atherosclerotic disease, established cardioembolic source, small-vessel occlusive disease, hypercoagulable disorder requiring anticoagulation or arterial dissection). Patients with history of MI or unstable angina, history of intracranial bleeding, pre-existing neurological disorders, left ventricular systolic dysfunction with aneurysm or akinesia, contraindications to antiplatelet therapy or underlying malignant disease. | Other likely cause of the ischaemic event. Non-eligible reasons for PFO closure included: migraine with/without aura, decompression illness, orthodeoxia-playpnoea syndrome, other desaturation syndrome, MI (presumed embolic), peripheral embolus, prior to neurosurgical procedure, high altitude pulmonary oedema, primary prevention (stroke). Other clinical history not part of eligibility criteria.  Two patients had 2 PFO closure procedures. |
| *Intervention* | | | | | | | |
| Device used | STARFlex (100%) | Amplatzer (100%) | Amplatzer (100%) | Amplatzer (58.7%)  Cardia Intrasept occluder (13.2%)  St Jude Premere (9.4%)  STARFlex (8.9%)  Occlutech Figulla (7.7%)  Cardia Atriasept (1.3%)  Gore (0.9%) | Gore HELEX septal occluder (38.7%)  Gore Cardioform septal occlude (61.2%) | Amplatzer (100%) | St Jude Amplatzer (54.9%)  Gore (30.2%)  Occlutech Figulla (12.7%)  Other including combination (2.2%) |
| *Demographics^*^* | | | | | | | |
| Age, mean (SD) [range], years | 46.3 (9.6)  [18-60] | 45.7 (9.7) | 44.3 (10.2) | 42.9 (10.1) | 45.4 (9.3) | 49 (15) | 43.7 (11.4) [17-82] |
| Male | 52.1% | 53.7% | 45.1% | 57.6% | 59.2% | 55.0% | 56.9% |
| Diabetes | *Not reported* | 6.6% | 2.5% | 1.3% | 4.1% | 10.0% | 2.8% |
| Hypertension | 33.8% | 31.7% | 24.0% | 11.3% | 25.4% | 20.0% | 11.1% |
| Previous MI | 1.6% | 1.0% | 1.5% | 0% | *Excluded from study* | *Excluded from study* | 2.2% |
| Previous history of arrhythmia | 5.8% | *Not reported* | *Not reported* | *Not reported* | *Not reported* | *Not reported* | 2.6% |
| Peripheral vascular disease | 1.1% | 1.0% | 1.5% | *Not reported* | *Not reported* | *Not reported* | 0.4% |
| Previous stroke | 72.6% | 100.0% | 80.9% | 100% | 91.2% | *Not reported* | 88.0% |
| Previous TIA | 27.4% | 0% | 16.2% | 0% | 8.8% | *Not reported* | 15.4% |
| Previous peripheral embolism | *Not reported* | *Not reported* | 2.9% | ? | *Not reported* | *Not reported* | *Not eligible for inclusion* |
| *Outcomes* | | | | | | | |
| Primary outcome | Composite of stroke or TIA | Composite of recurrent nonfatal ischemic stroke, fatal ischemic stroke, or early death after randomisation | Composite of death, non-fatal stroke, TIA, or peripheral embolism. | Fatal or non-fatal stroke. | Ischaemic stroke | Composite of stroke, vascular death, or thrombolysis in MI defined major bleeding | Composite of ischaemic/haemorrhagic/undetermined stroke, TIA, RIND and death |
| Mean follow-up (SD), years | *Not reported* | 2.6 (2.0) | 4.1 | 5.4 (1.9) | 3.2§ | 2.8§ | 2.0 (0.8) |
| Events | *Not reported* | 9† | 9 (7 confirmed after independent adjudication) | 0 | 6 | 0 | 38 |
| Event rate at 1 year | *Not reported* | 1.3% | *Not reported* | 0% | *Not reported* | *0%* | 3.2 [2.0 to 4.4]% |
| Event rate at 2 years | 5.8% | 1.6% | *Not reported* | 0% | *Not reported* | 0% | 4.6 [3.1 to 6.0]% |
| Event rate at 5 years | *Not reported* | 2.2% | *Not reported* | 0% | *Not reported* | *Not reported* | *Not reported* |
| Abbreviations: AF atrial fibrillation; CVA cerebrovascular accident; MDT multi-disciplinary team; MI myocardial infarction; PFO patent foramen ovale; RCT randomised controlled trial; RIND reversible ischaemic neurological deficit; SD standard deviation; TIA transient ischaemic attack; TOE transoesophageal echocardiography; TTE transthoracic echocardiography  * from closure group in RCTs  † intention to treat  § median follow-up  ⱡ study reported 10.6% experienced previous stroke (prior to index cryptogenic ischaemic stroke) and 11.6% experienced previous TIA (prior to index cryptogenic ischaemic stroke)  ^&^ follow-up of trial (not specific to closure group)  Footnote: Event rates reported in our study and the CLOSURE-1 study were from Kaplan-Meier analysis; event rates reported in the other RCTs were taken at review points. | | | | | | | |
