# Supplementary material for: Patent foramen ovale closure: A prospective UK registry linked to hospital episode statistics
Source: PLoS One. 2022 Jul 14;17(7):e0271117. doi: 10.1371/journal.pone.0271117 (PMC9282467; doi:10.1371/journal.pone.0271117)
Supplement: S1 Fig — (DOCX) [file pone.0271117.s007.docx]

Figure S1: Data flow describing linkage to HES

Excluded (multiple reasons may apply)

- 2 patients with identical records (non-unique)

- 11 patients with overlapping admissions

- 16 with no hospital admission +/- 7 days of PFO closure admission date

- 21 with no cardiac procedure coded (any OPCS4 code from “K” Heart chapter)

- 10 with gender mismatch

- 7 with age discrepancy larger than +/- 1 year

Excluded

- 25 type 2 opt-outs removed (those not wishing for their patient information to be used for purposes other than that of their individual care)

- 14 unmatched by NHS Digital

840 patients matched to HES and followed in HES & ONS

888 patients

5907 episodes of care identified from HES Admitted Patient Care

916 procedures

914 patients recorded in registry on 5th April 2018 and sent to NHS Digital for data linkage
