# Supplementary figures and images for: Patent foramen ovale closure: A prospective UK registry linked to hospital episode statistics
Source: PLoS One. 2022 Jul 14;17(7):e0271117. doi: 10.1371/journal.pone.0271117 (PMC9282467; doi:10.1371/journal.pone.0271117)

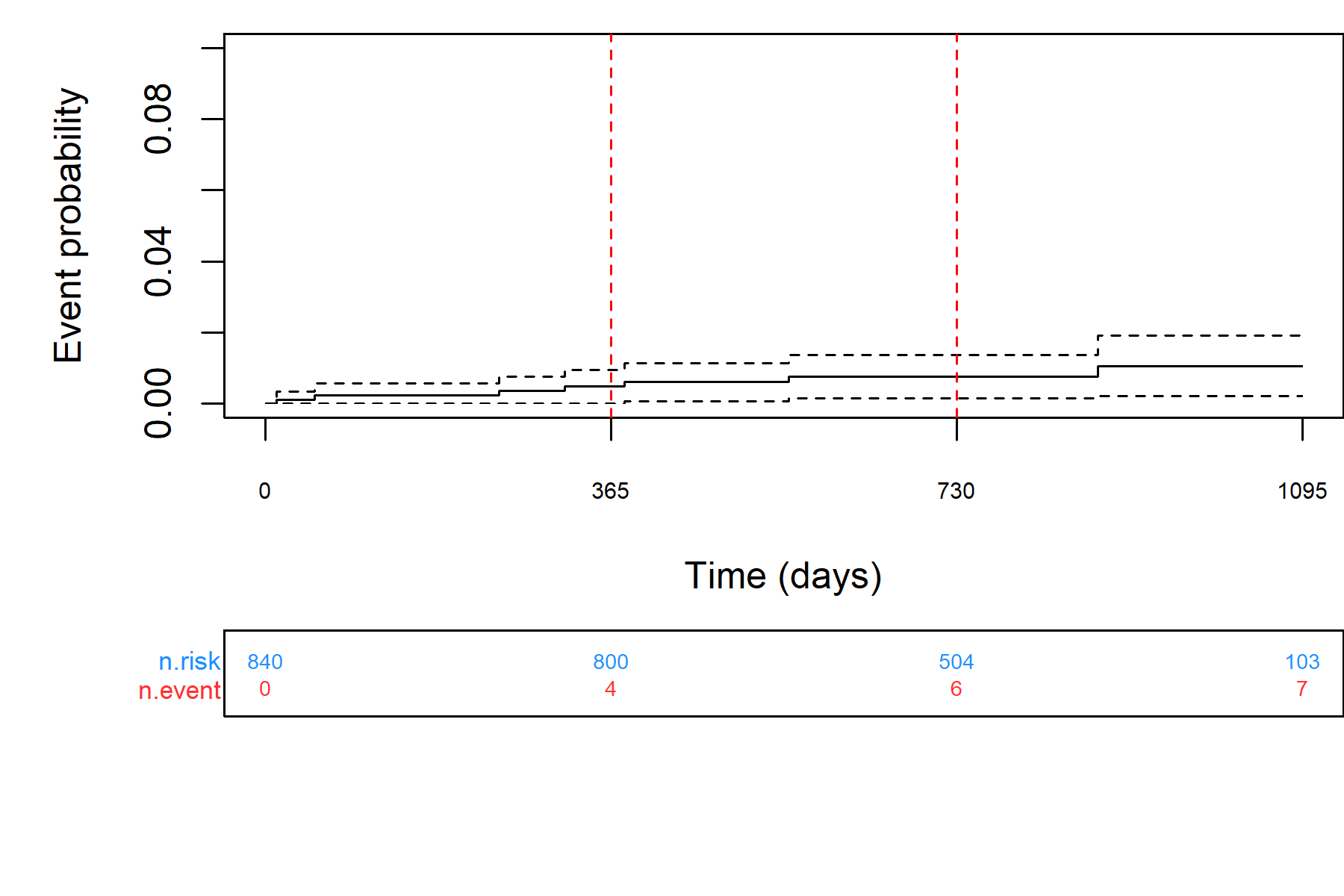

Supplement: S2 Fig — (TIFF) [file pone.0271117.s008.tiff]

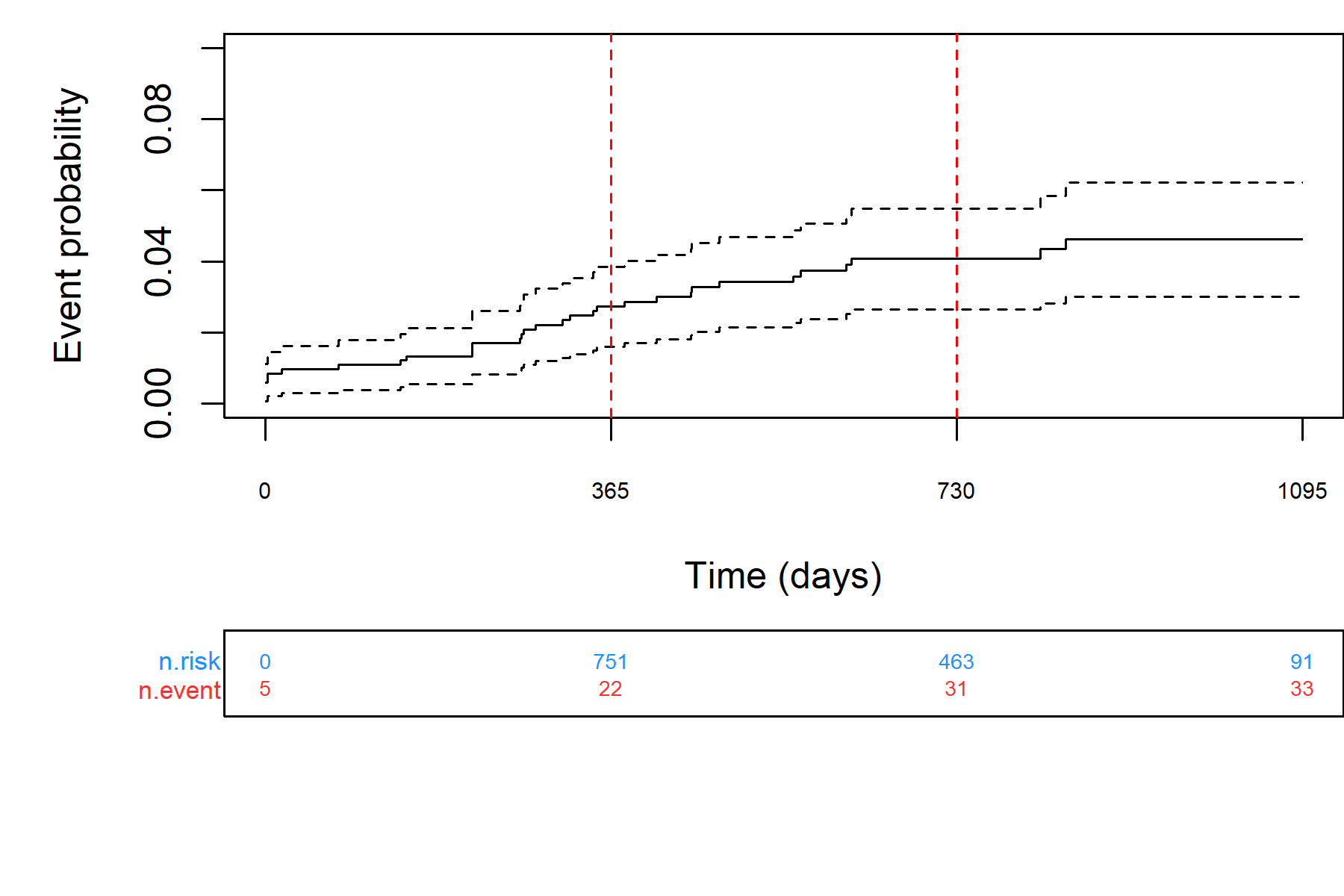

Supplement: S3 Fig — (TIFF) [file pone.0271117.s009.tiff]
